# Regulations Governing the Height of Houses

**Published:** 1885-02

**Authors:** 


Regulations Governing the Height of Houses.—The number of dwelling houses in Paris is about 90,000. The area of the whole city is twenty-five square miles, and the population over 2,000,000. A recent measure of the Council d'Etat ordains that henceforward no flats shall be less than eight feet high; that in streets twenty-five feet wide the height of the houses must not exceed forty feet; in streets between twenty-five and thirty-two feet wide the height must not exceed fifty feet; in streets between thirty-two and sixty-five feet wide the height must not exceed sixty feet; in streets above sixty-five feet wide the height must not exceed sixty-five feet, and no buildings are to have more than seven stories, all included.—*The Sanitarian.*

